# Patterns and functional implications of rare germline variants across 12 cancer types

**DOI:** 10.1038/ncomms10086

**Published:** 2015-12-22

**Authors:** Charles Lu, Mingchao Xie, Michael C. Wendl, Jiayin Wang, Michael D. McLellan, Mark D. M. Leiserson, Kuan-lin Huang, Matthew A. Wyczalkowski, Reyka Jayasinghe, Tapahsama Banerjee, Jie Ning, Piyush Tripathi, Qunyuan Zhang, Beifang Niu, Kai Ye, Heather K. Schmidt, Robert S. Fulton, Joshua F. McMichael, Prag Batra, Cyriac Kandoth, Maheetha Bharadwaj, Daniel C. Koboldt, Christopher A. Miller, Krishna L. Kanchi, James M. Eldred, David E. Larson, John S. Welch, Ming You, Bradley A. Ozenberger, Ramaswamy Govindan, Matthew J. Walter, Matthew J. Ellis, Elaine R. Mardis, Timothy A. Graubert, John F. Dipersio, Timothy J. Ley, Richard K. Wilson, Paul J. Goodfellow, Benjamin J. Raphael, Feng Chen, Kimberly J. Johnson, Jeffrey D. Parvin, Li Ding

**Affiliations:** 1 The McDonnell Genome Institute, Washington University in St. Louis, Forest Park Avenue, Campus Box 8501, St Louis, Missouri 63108, USA; 2 Department of Medicine, Washington University in St. Louis, Forest Park Avenue, Campus Box 8501, St Louis, Missouri 63108, USA; 3 Department of Genetics, Washington University in St. Louis, St Louis, Missouri 63108, USA; 4 Department of Mathematics, Washington University in St. Louis, St Louis, Missouri 63108, USA; 5 Department of Computer Science, Brown University, Providence, Rhode Island 02912, USA; 6 Center for Computational Molecular Biology, Brown University, Providence, Rhode Island 02912, USA; 7 The Ohio State University Comprehensive Cancer Center, Columbus, Ohio 43210, USA; 8 Siteman Cancer Center, Washington University in St Louis, St Louis, Missouri 63108, USA; 9 Department of Pharmacology and Toxicology, Medical College of Wisconsin, Milwaukee, Wisconsin 53226, USA; 10 Brown School Master of Public Health Program, Washington University in St Louis, St Louis, Missouri 63130, USA; 11 Department of Biomedical Informatics and, Ohio State University, Columbus, Ohio 43210, USA

## Abstract

Large-scale cancer sequencing data enable discovery of rare germline cancer susceptibility variants. Here we systematically analyse 4,034 cases from The Cancer Genome Atlas cancer cases representing 12 cancer types. We find that the frequency of rare germline truncations in 114 cancer-susceptibility-associated genes varies widely, from 4% (acute myeloid leukaemia (AML)) to 19% (ovarian cancer), with a notably high frequency of 11% in stomach cancer. Burden testing identifies 13 cancer genes with significant enrichment of rare truncations, some associated with specific cancers (for example, *RAD51C*, *PALB2* and *MSH6* in AML, stomach and endometrial cancers, respectively). Significant, tumour-specific loss of heterozygosity occurs in nine genes (*ATM*, *BAP1*, *BRCA1/2*, *BRIP1*, *FANCM*, *PALB2* and *RAD51C/D*). Moreover, our homology-directed repair assay of 68 *BRCA1* rare missense variants supports the utility of allelic enrichment analysis for characterizing variants of unknown significance. The scale of this analysis and the somatic-germline integration enable the detection of rare variants that may affect individual susceptibility to tumour development, a critical step toward precision medicine.

## Abstract (web summary)

Published sequencing data sets of cancer samples could be used to identify genetic variants associated with the risk of developing cancer. Here, Lu *et al*. analyse over 4,000 tumour-normal pairs to reveal variable frequencies of inherited susceptibilities across 12 cancer types and find enrichment of functionally validated missense variants of unknown significance.

## Graphical abstract

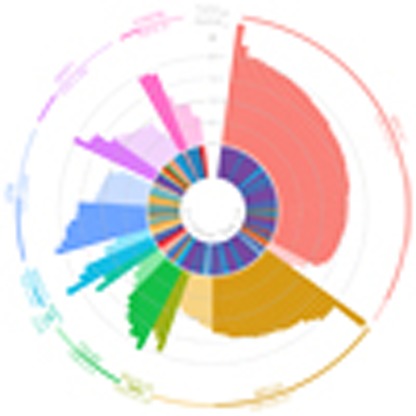

At least 3% of all cancer cases are thought to have a strong hereditary component, with large variation being found across cancer types^1^. For example, it was recently estimated that up to 20–25% of ovarian cancers are due to a germline loss-of-function variant in one of several genes that confer moderate-to-high risk^2^,^3^, while other cancer types (for example, lung) have strong environmental components with little evidence of genetic predisposition^4^. The absence of heritability in some cancers may be due to low or medium penetrance alleles^5^. Genome-wide association studies (GWAS) have been instrumental in identifying hundreds of common low-effect risk alleles across multiple cancer types^6^. The availability of large-scale normal and tumour-sequencing data from cancer cases now allows for discovery of rare variants influencing cancer susceptibility through analysis of both germline and somatic sequencing data.

Tumorigenesis is a complex process that often involves close interactions between germline and somatic variants. Their cooperation is best exemplified by the ‘two-hit hypothesis'^7^, in which a tumour suppressor gene is inactivated by the combination of an initial germline mutation of one allele, followed by the somatic inactivation of the other. Loss of heterozygosity (LOH), whereby the wild-type (WT) allele for a two-hit tumour suppressor is eliminated, has been implicated in many cancers^8^,^9^. Advancing our understanding of cooperative germline-somatic dynamics and their implications for tumorigenesis requires large cohort studies using sequencing data from both germline and somatic tissues, as well as new tools to reliably detect allelic loss.

We have previously reported that whole exome sequencing data can be successfully employed to identify both known high penetrance cancer genes in ovarian cancer, as well as new candidate predisposition alleles for downstream functional characterization^3^. Here we extend this work to 12 cancer types with the goal of describing the landscape of germline variants (truncation and missense) and analysing the effect of germline variants on somatic mutations using >4,000 cancer cases. Our analysis shows a diverse set of genes potentially contributing to predisposition with variable frequencies and levels. Stomach cancer has a relatively high rate of rare germline truncations, in large part due to frequent *PALB2* and *ATM* mutations. Genes and local hotspots of significant allelic enrichment within functional domains were discovered through integrating germline and somatic data. Germline and somatic integration sheds insights on genes influencing somatic mutation frequencies and genes/pathways involved in the entire life history of individual tumours. Experimental validation of 68 *BRCA1* variants, with 62 having previously unknown functional significance or not reported by the NHGRI Breast Cancer Information Core (BIC) database, identified 9 with complete or partial loss of homology-directed repair (HDR) function, further supporting LOH analysis results. Such discovery of new cancer susceptibility genes and functional characterization of variant alleles will be an important step towards generating an actionable catalogue for personalized treatment of cancer.

## Results

### Cancer types and sample characteristics

**Figure 1:**
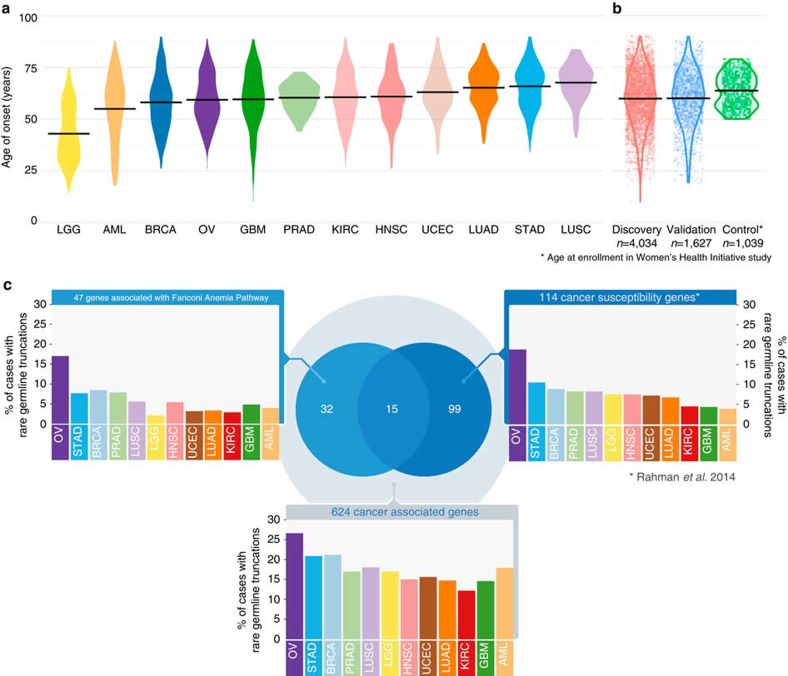
Characteristics of the data. Data are distributed by age, cancer, cohort and carrier frequency. (**a**) Age of onset by cancer type. Average age varies across cancer types, from 43 years in LGG to 67.7 years in LUSC. Note that LGG, LUAD and STAD show clear bimodal characteristics. (**b**) Age distributions for discovery, validation and control cohorts. (**c**) Comparison of cancer gene truncation carrier frequencies across 12 cancer types. The distribution of rare germline truncation variants for 12 cancer types (represented as the per cent of cases in each cancer type with rare germline truncation mutation) in 2 different groups of cancer-associated genes (labelled on top of each bar plot): 114 cancer susceptibility genes from Rahman *et al*.^1^ and 47 genes associated with the DNA repair (Fanconi Anaemia) pathway^3^. There are 15 genes common to both groups. The total number of unique genes from these 2 groups is 131.

We searched for candidate germline cancer predisposition variants in the exome sequence data from 4,034 cancer patients across 12 diverse cancer types: breast adenocarcinoma (BRCA), glioblastoma multiforme (GBM), head and neck squamous cell carcinoma (HNSC), kidney renal clear cell carcinoma (KIRC), acute myeloid leukaemia (AML), low grade glioma (LGG), lung adenocarcinoma (LUAD), lung squamous cell carcinoma (LUSC), ovarian carcinoma (OV), prostate adenocarcinoma (PRAD), stomach adenocarcinoma (STAD) and uterine corpus endometrial carcinoma (UCEC). The numbers of cases from each tumour type ranged from 178 (PRAD) to 770 (BRCA) and are listed in Table 1. Of the 3,548 TCGA cases with available ethnicity information, 88.1% were Caucasian (*n*=3,125), 6.3% were African American (*n*=225), 5.2% were Asian (*n*=183) and 0.4% (*n*=15) were American Indian/Alaska Native. Patients (*n*=3,827) were diagnosed between 10 and 90 years (mean 59.9±13.2 years) with LUSC and LGG having the highest and lowest mean ages, respectively (Table 1). The sex distribution is generally consistent with US general population cancer statistics for these malignancy types. Age of onset distribution is bimodal for LGG, LUAD and STAD, with some evidence of a bimodal distribution for OV, KIRC, HNSC and GBM. Distinct age of onset populations may indicate discrete mutational or disease processes (Fig. 1a).

Sequencing data for an additional 1,627 TCGA cases were collected for 10 out of the 12 cancer types (AML and STAD not included) for validating findings from the discovery cohort. In the validation cohort, 1,388 TCGA cases had available demographic information, of which 1,173 cases had ethnicity information, where 83.8% were Caucasian (983 out of 1,173), 12.79% were African American (150 out of 1,173), 2.98% were Asian (35 out of 1,173) and 0.4% (5 out of 1,173) were American Indian/Alaska Native/Native Hawaiian or Other Pacific Islander. Patients (*n*=1,388) were diagnosed between 19 and 90 years (mean 59.8±13.1 years), with LUAD and LGG having the highest and lowest mean ages, respectively (Fig. 1a and Table 1).

Sequencing data for samples from the National Heart Lung and Blood Institute (NHBLI) Women's Health Initiative Exome Sequencing Project (WHISP) were downloaded, processed and used for comparison of genetic variants to TCGA cancer cases. After extensive quality checks (see Methods), 1,039 Caucasians with an average age of 63.7±7.9 years (mean±s.d., range 50–79) were selected as controls for downstream burden test analyses (Fig. 1b). NHLBI variant calls for 6,503 samples (4,300 Caucasians and 2,203 African American) were also downloaded from the NHLBI Exome Variant Server (ESP6500SI-V2, http://evs.gs.washington.edu/EVS/) for additional comparative analyses.

### Landscape of germline truncation and missense variants

Germline variant calling was conducted using VarScan^10^, GATK^11^ and Pindel^12^ for TCGA discovery (4,034) and validation (1,627) samples and Women's Health Initiative (WHI) (1,039) controls. False-positive filters were applied to the intersected indel calls to ensure high quality for downstream analyses. Missense variants were further analysed by comparing with recurrent somatic mutation sites and IARC and ClinVar databases (Supplementary Note 1). Examination of coverages in the TCGA and WHI samples across the exome showed comparable depths, with averages of 115.3 and 106.2, respectively (see Supplementary Data 1). Specifically, there is a high positive correlation (Pearson correlation *R*=0.98) of the percentage of coding regions with at least 30 × coverage between WHI (70.8%) and TCGA (71.4%) samples across the 624 cancer genes selected based on several recent studies^13^,^14^,^15^,^16^,^17^ (Supplementary Fig. 1).

We identified 2,089 truncation variants (splice site, frameshift indels, nonstop and nonsense) in the TCGA discovery cohort in 624 cancer-associated genes (see Methods and Supplementary Data 2,3,4). We limited our analysis to variants whose minor allele frequency between our discovery data set and that of NHLBI Exome Sequencing Project (ESP) 6,503 was ≤0.05%, based on the distribution of minor allele frequencies across *BRCA1* and *BRCA2* truncations detected (Supplementary Fig. 2). After manual curation, we retained 838 truncation variants in 249 genes previously implicated in cancer (Supplementary Data 2); 69 of them with whole genome sequencing coverage have all been confirmed (Supplementary Data 4).

We conducted a more stringent investigation of the distribution of the rare truncation variants (MAF≤0.05%) across cancer types using two different gene sets: 114 well-known cancer susceptibility genes reported by Rahman *et al*.^1^ and 47 DNA repair genes associated with Fanconi Anaemia pathway^3^, with 15 overlapping between the two sets (Fig. 1c, and Supplementary Data 5 and 6). Examination of the 114 susceptibility genes^1^ revealed that ovarian (19%, 95% confidence interval (CI): 16–23%) and stomach (11%, 95% CI: 8–14%) cancers have the highest percentage of cases carrying rare truncation variants, while AML (4%, 95% CI: 2–8%) and GBM (4%, 95% CI: 3–8%) have the lowest number of such events (Fig. 1c). Ovarian (17%, 95% CI: 14–20%), prostate (8%, 95% CI: 4–12%) and breast (8%, 95% CI: 6–10%) cancers exhibit the highest percentage of cases harbouring rare truncations when the 47 DNA repair genes associated with Fanconi Anaemia pathway are included. Stomach cancer (8%, 95% CI: 5–11%) in the Fanconi Anaemia pathway-related genes also displayed relatively high truncation rates. Interestingly, LGG (2%, 95% CI: 1–5%) and KIRC (3%, 95% CI: 2–5%) have the lowest truncation rates in the Fanconi Anaemia pathway-related genes, consistent with the small numbers of somatic variants identified in these two cancer types.

### Genes significantly associated with cancer predisposition

**Figure 2:**
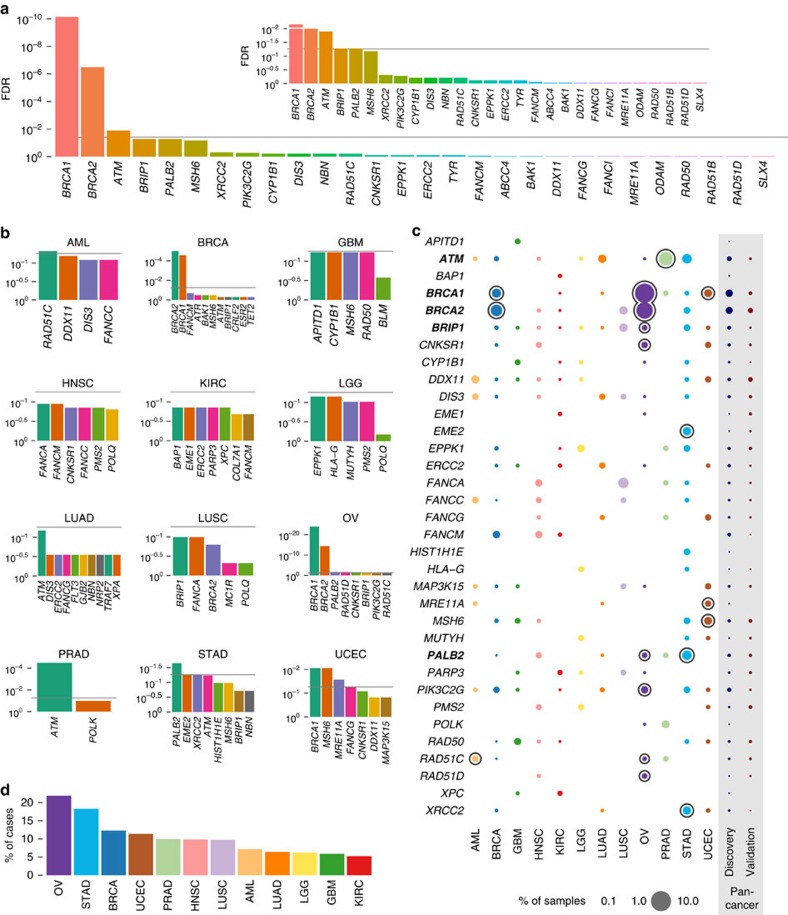
Burden analysis reveals distinct set of cancer susceptibility genes across 12 cancer types. A total of 34 genes-of-interest were identified by burden analysis by comparing the frequencies of rare truncation variants in Caucasian cancer cases (*n*=3,125) versus their frequencies in the WHI control population (*n*=1,039). Two oncogenes (*ABL2* and BCR) were omitted. (**a**) Significant genes across Pan-Cancer types. Data were analysed with the total frequency test (TFT) followed by false discovery rate (FDR) ranking. Dark horizontal line indicates the 5% FDR threshold, which is satisfied by five genes, including *BRCA1, BRCA2, ATM, BRIP1* and *PALB2*. Inset shows closer visual resolution. (**b**) Significant genes for specific cancer types. Each plot shows the top tested genes, by FDR, from the same TFT analysis procedure for all 12 individual cancer types. Eight genes in addition to the five shown in **a** are significant at the 5% FDR level from cancer-type-specific analysis. (**c**) Cohort frequencies of genes. Bubble plot shows frequency of rare truncation mutation as a percentage of cases in each cohort (all 4,034 cases included for frequency calculation). The *x*-axis denotes the test group of a specific cancer type, the Pan-Cancer discovery cohort (4,034) and the validation cohort (1,627). Genes found to be significant at 5% FDR using the Pan-Cancer discovery cohort are labelled in boldface. Rings indicate genes that are significant (TFT, FDR ≤5%) for a particular cohort on the *x*-axis. (**d**) Percentage of cases carrying rare truncation in the 34 genes-of-interest across 12 cancer types in the discovery cohort.

Out of 4,034 total discovery cases, 3,125 were identified as Caucasians based on reported clinical data. We performed burden analysis in Caucasians (3,125 cases versus 1039 WHI Caucasian controls, see Supplementary Data 7) using well-established methods^18^,^19^ (see Methods). To obtain the most comprehensive information, we also performed comparisons between the TCGA 4,034 cases and ESP 6,503 (downloaded variant calls, see Supplementary Data 8). We searched for genes displaying significantly higher rare truncation variant frequencies than the background rate derived from WHI 1,039 control set (see Methods) and identified 13 significant genes (false discovery rate (FDR) ≤5%) using total frequency test (TFT) calculations^19^, 5 from cross cancer-type analysis and an additional 8 from individual cancer-type analysis, with *BRCA1*, *BRCA2, ATM, BRIP1* and *PALB2* as the top 5 ranked genes associated in the Pan-Cancer analysis, and other genes including *CNKSR1*, *EME2*, *MRE11A*, *MSH6*, *PIK3C2G*, *RAD51C*, *RAD51D* and *XRCC2* associated with specific cancer types (Fig. 2a,b and Supplementary Data 7).

We detected 53 *BRCA1* rare truncation variants across 7 cancer types and 50 *BRCA2* rare truncation variants across 6 cancer types (Fig. 2c). As expected, most variants were detected in ovarian and breast cancer cases. However, seven *BRCA1* and six *BRCA2* germline truncations (MAF≤0.05%) were detected in other cancer types (three each in endometrial, stomach and lung cancers, two in kidney cancer and one each in prostate and head and neck cancers). The average age at diagnosis of *BRCA1* and *BRCA2* germline truncation carriers versus non-carriers was non-significantly younger for endometrial (52.7 versus 63.1), stomach (59.7 versus 66.1) and lung (63.0 versus 66.1) cancers, providing support that these variants may contribute to younger onsets of these cancer types, though additional data is required for confirmation and to reach statistical significance. We also observed 32 truncations in *BRCA1* and *BRCA2* interacting proteins: *PALB2* (*n*=12, 4 in stomach, 3 in ovarian, 2 in head and neck and each in breast, lung and prostate cancers), *BRIP1* (*n*=16, 3 each in breast, ovarian and lung, 2 in stomach, 1 each in GBM, HNSC, KIRC, LGG and UCEC), and *BAP1* (*n*=2, in kidney) and *BARD1* (*n*=2, 1 each in PRAD and BRCA). *ATM* was the third most significant gene and the third highest in number of rare truncation variants; a total of 28 were found in *ATM* (23) and its homologue, *ATR* (5) (Supplementary Data 7). Our study bolsters evidence for previously claimed *ATM/ATR* associations with breast cancer with observations of 4 *ATM* and 4 *ATR* truncations in breast cancer cases. Notably, 19 *ATM* truncations were also detected in other cancer types, mostly in lung, stomach and prostate cancers, the respective fractions of cases being 1.1% (5 out of 462 cases), 1.2% (4 out of 321 cases) and 3.4% (6 out of 178 cases). These fractions are all higher than the observed 0.5% in breast cancer. Both *ATM* and *ATR* are serine/threonine protein kinases that act upstream from cell cycle check point proteins *CHEK2* (6) and *CHEK1* (1), respectively. The rest of the significant genes were linked to various DNA repair pathways. For example, *MSH6* (11) is a component of the mismatch repair pathway and *XRCC2* (7), *RAD51C* (6), *NBN* (9) are all part of the DNA double-strand repair pathway. *ERCC1* (3) and *ERCC2* (10) are involved in transcription-coupled nucleotide excision repair. Four rare truncations (two in LGG) were also found in *MUTYH* (a mutY homologue), involved in oxidative DNA damage repair (Supplementary Data 7).

We also sought to identify genes enriched for truncations that were significantly associated with single or a subset of cancer types. *RAD51C* was found to be significant in OV and significant and top ranked in AML, while *PALB2* truncations were associated with STAD and OV (Fig. 2b). *PMS2*, involved in colorectal^20^ and endometrial cancer^21^ predisposition, showed suggestive associations with HNSC (TFT, FDR=14%) and LGG (TFT, FDR=10%) in the discovery set, but did not reach the 5% FDR threshold (Fig. 2b). Significant enrichment of *MSH6* (6 were close to the C terminus of the protein) and *MRE11A* truncations were found in UCEC. Other notable genes that were significant in a specific cancer type included *EME1* in KIRC and *FANCM* in BRCA (Fig. 2b and Supplementary Data 7). Notably, we observed several novel associations between specific cancer types and genes, including *RAD51C* in AML, *ATM* in PRAD, *PALB2* and *EME2* in STAD.

To further evaluate these findings, we investigated rare truncations in those 13 significant genes, as well as an additional 21 suggestive genes having FDR ≤15% (TFT) using another independent set of 1,627 cancer cases from 10 of the 12 cancer types (see Methods). Our analysis showed that additional rare truncations (MAF≤0.05%) were identified in 26 out of these genes in the validation set (Supplementary Data 3). The overall frequencies correlate positively (Pearson coefficient of 0.6167, Supplementary Fig. 3). Notably, 10 rare *PMS2* truncations were found in the validation set, with 4 from UCEC, 2 each from LUAD and LUSC and 1 each from BRCA and PRAD; these observations confirm the significance of *PMS2* in susceptibility and broaden its role in cancer types not previously implicated. Another example is *XPA* detected as significant using the discovery cohort and confirmed by the identification of two additional rare truncations (E111* and V244fs) in prostate cancer using the validation cohort. Although three additional *ATM* rare truncations were found in BRCA and GBM in the validation cohort, no events were detected in LUAD and PRAD, two cancer types with significant results in the discovery cohort. Overall, our results from the validation cohort strengthen provisional conclusions derived in the discovery phase, but also indicate that larger cohorts are required for accurately assessing frequencies of germline mutations, as well as detecting low frequency events in individual cancer types.

### LOH analysis of rare truncation and missense variants

While burden analysis can identify genes with significant enrichment of rare truncations, association studies have limitations, specifically with respect to inference about the functional implications of specific variants. LOH analysis can uncover heterozygous germline variants that are under potential selection in the tumour, one of the key indications being increased VAF in the tumour sample. With no LOH, it would be expected that the VAF detected in tumour relative to the normal tissue-derived DNA would be 1, while with complete LOH the VAF ratio would be 2. Because tumour samples are not completely free of normal tissue and can exhibit clonal heterogeneity, evidence for LOH is increasingly strong for VAF ratios approaching 2. The combined use of burden tests that can narrow the search space for germline variants of functional importance with LOH analysis can solidify support for both putative genes and specific variants involved in cancer susceptibility.

**Figure 3:**
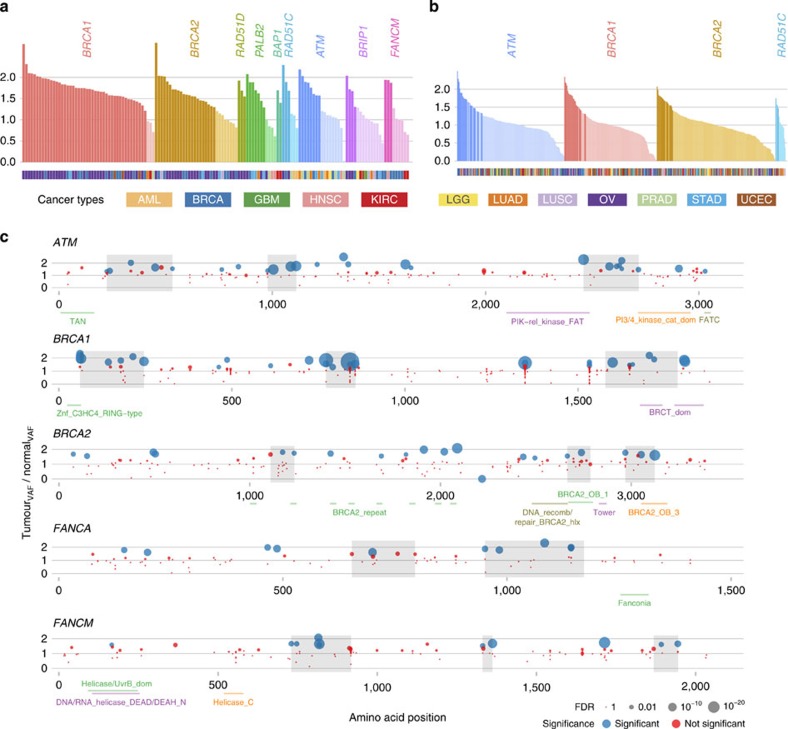
Analysis of loss of heterozygosity in rare truncation and missense variants. (**a**) Bar plot shows individual truncations from nine genes (FDR shown) with lengths representing ratios of tumour-to-normal variant allele fractions (that is, the fraction of reads containing the variant allele). Statistically significant events, defined as FDR≤5%, are shaded boldly, while non-significant events are muted, with colours corresponding to genes. Cancer source of each truncation is shown underneath, for example, most *BRCA1* variants occur in ovarian and breast cancers and all *BAP1* variants in KIRC. (**b**) Bar plot for individual missense variants from four genes having elevated frequencies of such variants that show very significant LOH, that is, at the 1% FDR level. (**c**) Dot plot shows individual missense variants where abscissa and ordinate are amino acid positions and the ratio of tumour-to-normal variant allele fraction, respectively. Blue and red indicate significant (FDR ≤5%) and non-significant events, respectively, with size of dots proportional to negative log of the FDR. Annotated domains from the PFAM database are aligned with position, while shaded areas indicate ‘hotspot' regions where variants having significant LOH cluster more than the rate explainable by chance. Plots are shown for *ATM*, *BRCA1*, *BRCA2*, *FANCA* and *FANCM*.

With respect to genes, we first tested the expanded list of 34 significant or nearly significant genes (known and likely oncogenes excluded) in burden analysis (see Methods) for evidence of somatic loss of the WT allele. A total of seven genes, *BRCA1, BRCA2, RAD51D, PALB2, RAD51C, ATM* and *BRIP1* were significant (FDR ≤5%) along with two genes (*BAP1* and *FANCM*) near significance (Supplementary Data 9 and 10, and Figs 2 and 3a). Consistent with expectations, *BRCA1* and *BRCA2* had the highest percentage of significant variants demonstrating LOH (44 of 48 (92%) and 21 of 30 (70%), respectively). Other genes demonstrating variants with LOH include: *PALB2*, which functions in maintenance and repair and cooperates with *BRCA2* (ref. 22) (5 significant truncation mutations of 11, 45%); *ATM*, which is activated by double-strand breaks (8 of 17 significant, 47%); *BAP1*, a transcriptional repressor involved in BRCA1-mediated cell growth suppression^23^ (2 of 2, 100%); and *FANCM*, which plays a role in DNA repair^24^ (3 of 9, 33%). In all, 99 of 264 (38%) truncation variants showed significant LOH. It is worth noting that although LOH in cases with *BRCA1* and *BRCA2* truncations mutations were largely restricted to OV and BRCA, the majority of LOH truncations in other genes (for example, *ATM*, *PALB2*, *BAP1*, *FANCM*) were found across cancer types (Fig. 3a).

We further compared VAFs of missense variants in the seven significant LOH genes above, finding that four in *BRCA1*, *ATM*, *BRCA2* and *RAD51C* are significant. This underscores both our findings from rare truncation analysis (Supplementary Data 11 and 12, and Fig. 3b) and the potential importance of missense events in cancer. The significant missense VAFs in these genes range from 13 to 23% (Fig. 3b), while other genes average 9%. Of all individual missense events, 173 of 1,170 (11%) showed significant LOH (FDR ≤1%) (Supplementary Data 12). Significant events for *ATM* and *BRCA1* were concentrated in BRCA, HNSC and OV, while *RAD51C* did not show preference (Fig. 3b). Of note, our LOH analysis identified G245V in *TP53* as highly significant (FDR=1.18e-07) although no rare *TP53* truncations were found.

To further investigate the effect of missense events on cancer susceptibility, we sought to determine whether there are any larger informative patterns associated with their LOH, specifically whether the significant instances of LOH spatially cluster in or near specific protein regions/domains. Indeed, analysis shows statistically significant difference in spatial clustering, further supporting the mechanistic roles of these variants in cancer (Fig. 3c). For example, there is a strong grouping of variants (FDR=0.34%) that overlaps both a kinase-like and a PIK kinase domain near the end of *ATM*, which participate in chromosome maintenance and repair. We also found clusters overlapping the BRCT (FDR=5%) and RING domains (FDR=0.39%), which participate in the DNA repair functionality of *BRCA1*. Two *BRCA2* clusters (FDRs=6.5% and 8.9%) in the oligonucleotide/oligosaccharide binding motif (OB fold) domains, important in the DNA damage response, are near significant (Supplementary Data 13).

### Somatic and germline interactions and clinical associations

**Figure 4:**
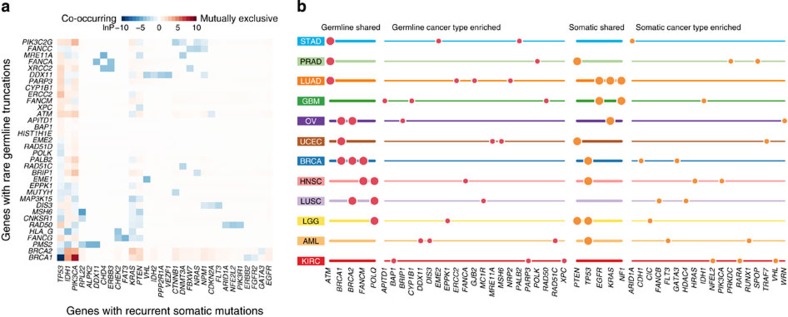
Molecular interactions between rare germline variants and somatic mutations within and across cancer types. (**a**) Heatmap demonstrates the significance of interactions between 34 burden test significant genes and 54 cancer-associated genes (top 30 are shown) with recurrently mutated somatic variants across cancer types. Red–white colour scale and blue–white colour scale depict the negative log of *P*-value for mutual exclusivity and co-occurrence, respectively. Both are based on the MuSiC permutation test (*n*=10,000). (**b**) Abacus plot displays the distribution of significant, mutually exclusive rare germline variants and somatic mutations across all 12 cancer types. Unique combinations of germline and somatic variants contribute to the development of individual cancer types. Bigger dots indicate recurrent genes across cancer types, while smaller dots indicate cancer-type-enriched genes.

We followed stringent filtering strategies^13^ for standardizing specificity across the Pan-Cancer somatic variant calls for 3,368 cases in this study (Supplementary Data 14). We first used MuSiC^25^ to search for genes demonstrating co-occurring or mutually exclusive germline and somatic mutations (Fig. 4a,b and Supplementary Data 15 and 16). Our Pan-Cancer analysis using 34 burden test genes-of-interest and 54 cancer-associated genes with recurrently mutated somatic variants (frequency ≥5 across cancer types) detected significant mutual exclusivity between *BRCA1*/*BRCA2* germline truncations and *IDH1* somatic mutations, which is likely confounded by cancer-type specificity: *BRCA1*/*BRCA2* germline truncations were most prevalent in BRCA and OV, whereas *IDH1* somatic variants are mostly found in AML, GBM and BLCA. To mitigate the cancer-type-specific effect, we investigated co-occurrence and mutual exclusivity within each cancer type (requiring recurrently mutated somatic variants with frequency ≥2 across cancer types) (Supplementary Data 16). Notably, *ATM* germline truncations were found to be mutually exclusive of *TP53* somatic mutations in LUAD (permutation test, *P*=0.041), consistent with the paradigm that ATM activates TP53 to trigger apoptosis^26^ and the need to disrupt only one gene to confer an anti-apoptotic effect. As expected, we also observed co-occurrence of *BRCA1* germline truncations and *TP53* somatic mutations in BRCA (permutation test, *P*=0.012)^27^, as well as mutual exclusivity between *BRCA1*/*BRCA2* germline truncations and *PIK3CA* somatic mutations in BRCA (permutation test, *P*=0.01 and *P*=0.03). *BRCA1* germline truncations have previously been reported to be associated with the basal subtype breast cancer^28^, which tends to exhibit a molecular profile similar to ovarian cancer^29^. Our findings are consistent with the association between basal subtype breast cancer and frequent *TP53* and infrequent *PIK3CA* mutations^30^. In addition, we also observed a co-occurrence of *BRCA2* germline truncations and *TP53* somatic mutations in ovarian cancer, as expected. Our data suggest that the combinational effects of *BRCA1*/*BRCA2* germline mutations, along with the high frequency of LOH events and somatic *TP53* mutations result in aggressive basal subtype breast cancer and ovarian cancer.

**Figure 5:**
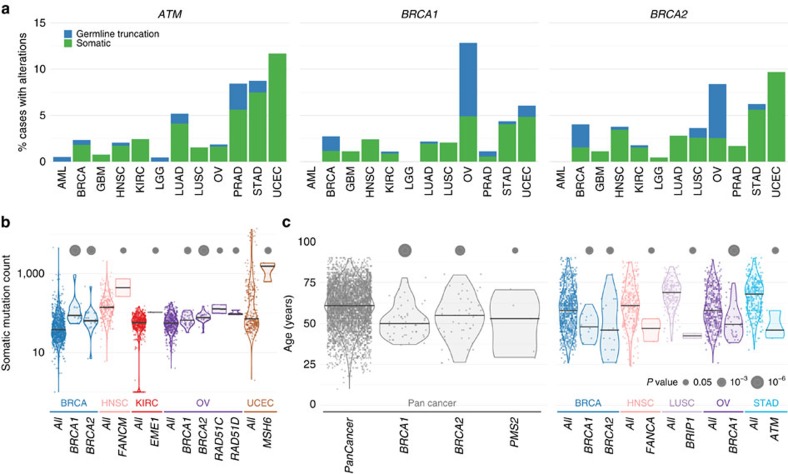
Germline variants correlate with somatic mutations and age at diagnosis. (**a**) Barplot illustrates the distribution of *BRCA1*, *BRCA2* and *ATM* somatic and germline mutations across cancer types. (**b**,**c**) Panels display genes significantly correlated with somatic mutation frequency and younger age of onset in different cancer types and in Pan-Cancer. The width of the shape indicates the density, and the horizontal line indicates the median. *P* value is calculated by the Wilcoxon rank-sum test and is indicated by the size of the uppermost circles.

Interestingly, the distribution of *BRCA1*, *BRCA2* and *ATM* rare germline truncations with their somatic mutations across cancer types varies with the high frequency of *ATM* in prostate, lung and stomach cancers, and *BRCA1* and *BRCA2* germline events in ovarian and breast cancers (Fig. 5a and Supplementary Data 17). Collectively, these analyses show distinct combinations of germline and somatic mutations contribute to the development of individual cancer types.

We also examined germline variants having significant impact on carriers' somatic mutation frequencies. Analysis of the expanded 34 burden test genes revealed that patients with germline *BRCA1* and *BRCA2* truncations had significantly higher somatic mutation frequencies than cases without such changes in both breast and ovarian cancers (Fig. 5b and Supplementary Data 18). Since the correlation between *BRCA1/2* germline and higher somatic mutation rate may be characteristic of the basal subtype breast cancer, we compared the mutation frequency of basal cases with *BRCA1/2* germline truncation to basal cases without *BRCA1/2* germline truncation and found the former have significantly higher mutation rate (Supplementary Fig. 4, Wilcoxon rank-sum test, *P*=9e-4).

In addition, *RAD51C* and *RAD51D* germline truncations are positively correlated with increased somatic mutation frequencies in ovarian cancer. *FANCM* and *EME1* germline truncations are positively correlated with increased somatic mutation frequencies in HNSC (Wilcoxon rank-sum test, *P*=0.046) and KIRC (Wilcoxon rank-sum test, *P*=0.027), respectively. In UCEC, *MSH6* germline truncations are found to be significantly associated with higher mutation frequencies, as expected (Wilcoxon rank-sum test, *P*=0.014) (Fig. 5b and Supplementary Data 18). Further, 81 cases carried *MSH2* germline variants (MAF≤0.05%, including 1 truncation variant), and they also showed higher somatic mutation frequency (Wilcoxon rank-sum test, *P*=3.63e-03).

The joint analysis of all 12 cancer types including cancer type as a covariate identified *BRCA1, BRCA2* and *PMS2* as having strong correlations with a younger age of onset (*P*=5.20e-07, 2.04e-04 and 0.049, respectively; MuSiC GLM analysis, Fig. 5c and Supplementary Data 19). Analysis of individual cancer types revealed significant early onset for germline truncations of *FANCA* in HNSC, *BRIP1* in LUSC and *ATM* in STAD (Fig. 5c and Supplementary Data 20). Not surprisingly, we found that germline truncation variants in 47 Fanconi Anaemia genes and 114 cancer susceptibility reported in Rahman *et al*.^1^ were significantly enriched in younger patients according to Wilcoxon rank-sum testing (*P*=1.08e-03 and 1.38e-04, respectively).

### Functional validation of *BRCA1* missense variants

To investigate the effect of missense variants on *BRCA1* function and evaluate LOH analysis for missense variants, 68 variants were selected based on MAF and protein domains for functional validation using the HDR assay^31^ (see Methods and Supplementary Data 21); 47 of them had previously been assigned as variants of unknown clinical importance in the NHGRI BIC database and 15 variants were not reported at all in BIC. One known deleterious truncation mutation in the carboxyl terminus of the BRCA1 protein Q1779fs and three other truncations—E1250*, E1415fs and E23fs—discovered in UCEC were also included in the experiment. We successfully introduced 68 missense variants and 4 truncation variants into full-length *BRCA1* expression plasmid pcDNA-5′HA-BRCA1 for the *in vitro* HDR assay as previously described^31^,^32^ (Supplementary Data 21). All mutant constructs were confirmed by sequencing and protein expression (Supplementary Fig. 5) and tested in triplicate using the *in vitro* assay. The percentages of cells showing green fluorescent protein (GFP) expression were normalized to homologous recombination levels observed in cells depleted of endogenous BRCA1 and rescued by transfection of the WT BRCA1 expression vector (see Methods).

**Figure 6:**
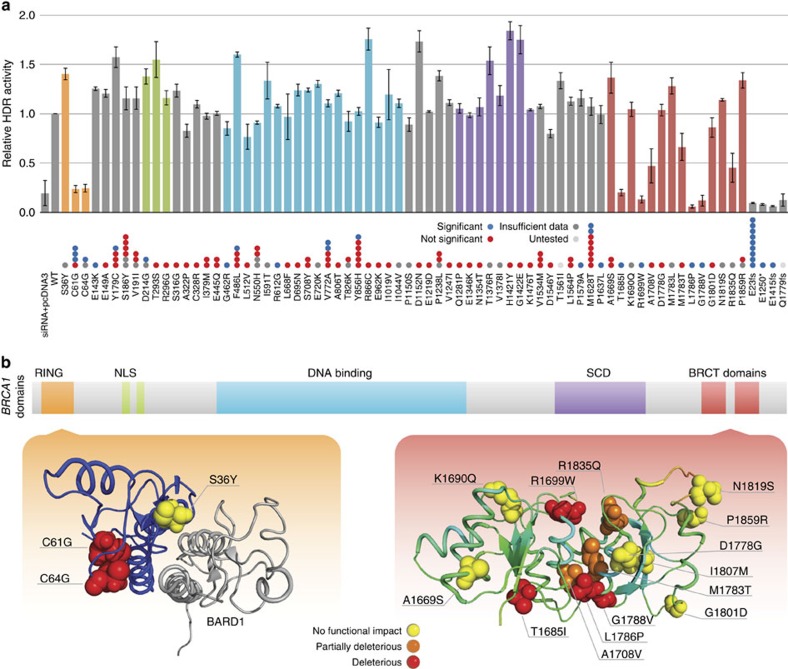
Functional validation of *BRCA1* missense and truncation variants. (**a**) 68 rare missense and 4 truncation variant sites were tested by HDR assay. All samples were depleted of endogenous BRCA1 by transfection of a siRNA targeting the 3′-untranslated region. Indicated in the legend are the plasmids transfected to test for rescue of BRCA1 activity. ‘pcDNA3' is empty vector and ‘WT' represents wild-type BRCA1 plasmid. The *y*-axis denotes the HDR activity relative to the wild-type BRCA1 protein. Error bars depict s.d. from the mean. Dots on the *x*-axis represent LOH status, each dot corresponding to one case. Blue, red, dark grey and light grey denote statistical significance, non-significance, unknown LOH (due to lack of sufficient coverage) and untested, respectively. Variants in different functional domains are indicated with colours as follows: orange, RING domain; green, nuclear localization signal (NLS); blue, DNA-binding region; purple, a SQ/TQ cluster domain (SCD); and red, *BRCA1* C-terminal domain (BRCT). All the HDR assays were tested in triplicate. (**b**) Crystal structure of the BRCA1 RING (left) domain in complex with the BARD1 RING domain (labelled in grey) and BRCT domain (right panel) are displayed, with HDR-defective variants labelled in red and partial HDR-defective variants tagged in orange. Variants in yellow are functional in the HDR assay.

Among all tested variants, all four truncations (three from UCEC) and six missense variants retained less than 30% of homologous recombination activities relative to WT *BRCA1*, and are therefore considered HDR-defective (Supplementary Data 22). These missense variants included C61G (observed in four cases), C64G (two cases), T1685I (one case), R1699W (two cases), L1786P (one case) and G1788V (one case); all of them showed significant enrichments in the tumour samples based on LOH analysis (Fig. 6a). Comparative analysis of RNA-seq data from two carriers and four non-carriers suggests C64G is in fact a variant affecting splicing (Supplementary Fig. 6), consistent with a previous report^33^, and our results suggest that should some of the C64G mRNAs be properly spliced the protein is not active in DNA repair. Of particular interest, L1786P, identified and validated as HDR-defective in our study, has not been previously designated as pathogenic, despite observations in two previous studies^34^,^35^. Our analysis of the crystal structure of the BRCT domain showed that the substitution of leucine with proline in L1786P will likely result in the termination of the alpha helix structure, which may cause the loss of *BRCA1* HDR function. Interestingly, additional three variants, A1708V, M1783T and R1835Q (from one patient each) consistently displayed less than 70% HDR function in comparison to WT *BRCA1* (partial HDR-defective, Fig. 6a); all three had previously been designated as variants of unknown significance in the BIC database. It is worth noting that A1708V and R1835Q were found in male patients with kidney and stomach cancers, respectively; both developed cancers at age of 48. A1708V has previously been characterized as a low-to-moderate risk variant^36^ and R1835Q has been identified in a Malay population of early-onset breast cancer patients with a personal or family breast cancer history^37^. One endometrial cancer patient harbouring M1783T was diagnosed at age of 65. The BRCA1 protein harbouring this variant was previously shown to possess enhanced protease sensitivity^38^. Further, our analysis shows that all seven HDR-defective or partial defective missense variants from the BRCT domain are either positioned in the centre of the structure or on the surface responsible for protein–protein interactions, while the 5 HDR-WT variants from the BRCT domain tested are mapped to the periphery of the structure (Fig. 6b). In addition, these nine HDR-defective (or partial HDR defective) missense variants are mutually exclusive to *BRCA1* somatic mutations and germline truncation variants (Supplementary Data 2 and 14).

Using the systematic *BRCA1* missense variant validation data, we evaluated the prediction power of LOH analysis for identifying candidate variants of functional relevance. Without LOH analysis filtering, we observed a rate of 4.7% (3 of 64 validated), but *BRCA1* validation of candidates filtered through LOH was increased to 38.1% (8 of 21) (Supplementary Data 23). The significant difference (*P* value=0.0004, Fisher's test) suggests LOH offers an effective sieve for candidates, which in this case gives an estimated enrichment factor of eightfold.

## Discussion

This study of over 4,000 cancer cases is the largest integrated analysis of germline and somatic variants to date. Our systematic analysis indicated that an estimated 18% of cancer cases from the TCGA cohort had ≥1 rare truncations in 624 genes associated with cancer. Further, there was significant enrichment of rare truncation variants in 13 genes and suggestive evidence of increases in 21 more, comprising 8.3% (333 out of 4,034) of TCGA cancer cases.

We observed several significant associations in specific cancer types: *RAD51C* in AML, *ATM* in PRAD and *PALB2* in STAD. Across cancer types, a higher percentage of breast and ovarian cancer cases were identified as having rare truncation variants in cancer genes versus other cancer types, due predominantly to high frequencies in *BRCA1/2*. The percentage of breast and ovarian cancer cases carrying *BRCA1/2* germline truncation variants in the TCGA cohort was 4.4% and 11.6%, respectively, consistent with previous reports^39^,^40^,^41^,^42^. Interestingly, stomach cancer has the second highest percentage of rare truncations in 114 genes previously reported^1^, largely due to the contributions from *ATM*, *BRIP1*, *PALB2*, *XRCC2* and others. In contrast, for KIRC and GBM, truncation variants in the 34 associated germline genes were uncommon, identified in only less than 6% of cases (Fig. 2d). These results contribute to our understanding of the genetic architecture of cancers, complementing the known effect of common and tagged variants from array-based studies^43^, as well as the estimate of overall heritability from twin studies in multiple cancer types^44^.

Our results indicated that germline truncation and missense variants in several genes were under selection in the tumour, with *ATM, BRCA1, BRCA2* and *RAD51C* determined as significant from both truncation and missense analyses and *BAP1, BRIP1, FANCM, PALB2* and *RAD51D* from truncation analysis alone. As a proof-of-concept, we performed functional validation for 68 *BRCA1* missense variant sites using HDR assay; our experimental efforts identified 9 variants from 14 patients with complete or partial defective HDR function and validated our LOH analysis for effective enrichment of variants under functional selection (an estimated eightfold enrichment in *BRCA1*).

More importantly, our integrated germline and somatic study identified *BRCA1*, *BRCA2*, *RAD51C*, *RAD51D*, *FANCM*, *EME1* and *MSH6* germline truncations significantly associated with increased somatic mutation frequencies in specific cancer types, suggesting that germline defects in DNA repair expand to the somatic level. Further, our search for co-occurring or mutually exclusive germline truncation/somatic mutations across 12 cancer types revealed a number of important insights in terms of genes and pathways involved including: (1) the association between germline *BRCA1/2* germline truncations and frequent *TP53* and infrequent *PIK3CA* somatic mutations confirm breast cancer clinical subtype classification; and (2) *ATM* as a *bona fide* (third frequently truncated) susceptibility gene demonstrated by both burden and LOH analyses is the only common gene highly mutated at both germline and somatic levels.

Although our study has been revealing at a genetic level, we are mindful of the limitations of the TCGA data set, including the lack of detailed family history information that would further inform the potential pathogenicity of germline variants. Despite the large sample size overall, our inferences are limited for specific cancer types because of small case numbers. In addition, the vast majority of TCGA cases in our sample set were of European background, emphasizing the need for the development of a reference source of genomic data on germline cancer predisposition variants from ancestrally diverse population groups. Nonetheless, this study is the largest to date that has integrated somatic and germline alterations to identify important genes across 12 major types contributing to cancer susceptibility and our results provide a promising list of candidate genes for definitive association and functional analyses. The combination of high throughput discovery and experimental validation should identify the most functionally and clinically relevant variants for cancer risk assessment.

## Methods

### Access and inclusion

Approval for access to TCGA case sequence and clinical data was obtained from the database of Genotypes and Phenotypes (dbGaP) (document #3281 Discover germline cancer predisposition variants). We selected a total of 4,034 discovery cases and 1,627 validation cases with germline and tumour DNA sequenced by exome capture followed by next-generation sequencing on Illumina or SOLiD platforms. All cases met our inclusion criteria of 50% coverage of the targeted exome having at least 20 × coverage in both germline and tumour samples.

### Control cohort

NHLBI variant calls for 6,503 samples (2,203 African-Americans and 4,300 European-Americans unrelated individuals) were downloaded from the NHLBI GO ESP, Seattle, WA (http://evs.gs.washington.edu/EVS/; accessed on 26 August 2013). For comparative analysis, all ESP variants were filtered for <0.1% total MAF to minimize false-positives. For the WHISP sample set (*N*=1039) as part of the NHLBI ESP cohort, we performed variant analyses using methods described in the following section. All variants were processed using the same tools as for the TCGA cohort. dbGaP accession ID for NHLBI ESP is phs00281.

### Germline variant calling and filtering

Sequence data from paired tumour and germline samples were aligned independently to GRCh37-lite version of the human reference using BWA v0.5.9 and de-duplicated using Picard 1.29. Germline SNPs were identified using Varscan (version 2.2.6 with default parameters except –min-var-freq 0.10--*P* value 0.1--min-coverage 8–map-quality 10) and GATK (revision5336) in single-sample mode for normal and tumour BAMs. For breast and endometrial cancer samples, we also used population-based methods, but found differences to be minimal. Germline indels were identified using Varscan 2.2.9 (with default parameters except --min-coverage 3–min-var-freq 0.2–*P*-value 0.10–strand-filter 1–map-quality 10) and GATK (revision5336, only for AML, BRCA, OV and UCEC) in a single-sample mode. We also applied Pindel (version 0.2.4 ×, 8 May 2013; window-size 1) on each pair of tumour and germline sequencing data (for some samples, multiple normal files are used if available) for indel prediction. For the analysis, we preset the insertion size to be 500 if this information was not provided in the BAM header.

For each cancer type, all variants were limited to coding regions of full length transcripts obtained from Ensembl release 70. In addition to the coding regions, the two base pairs flanking each exon that cover splice donor/acceptor sites were included and annotated with reference to the distance to the nearest coding exon (e). Single nucleotide variants (SNVs) were based on the union of GATK and VarScan. They were subsequently processed through our in-house false-positive filter (with default parameters except--min-homopolymer 10). We required that indels were called by at least two out of three callers (GATK, Varscan, Pindel) when all three callers were applied. In addition we also included Pindel unique calls (at least 30 × coverage and 20% VAF). All combined indels were then processed through our false-positive filter (with default parameters except--min-homopolymer 10 –min-var-freq 0.2 --min-var-count=6). We then applied additional annotation and minor allele frequency filters as previously reported^3^.

The predictions for 4,034 TCGA cases consist of 2,709,906 variants (1,655,391 missense, 947,045 silent, 36,009 nonsense, 18,693 splice site, 2,041 nonstop/readthrough, 30,508 frameshift indels and 20,219 in-frame indels) with minor allele frequency ≤1% in 1000 Genomes, ESP 6,503 data set, Discovery 4,034 cohort and additional annotation filters as previously reported^3^; of these, 1,842,459 variants were from 3,125 Caucasian TCGA cases. Using the same processing for the 1,039 WHI Caucasian controls, we identified 516,219 variants, consisting of 319,698 missense, 176,862 silent, 6,274 nonsense, 3,541 splice site, 355 nonstop/readthrough, 6,101 frameshift indels and 3,568 in-frame indels.

### Cancer-associated genes

A total of 624 candidate cancer-associated genes were compiled from 9 sources, including recently published large-scale cancer studies, publicly available screening panels and unpublished preliminary analysis of publicly available data sources. We retained 204 genes shared across at least 2 of the 9 sources and a literature search was conducted to identify evidence supporting inclusion of any remaining unique genes. A subset of 518 genes originated from recent publications, including 294 genes from Frampton *et al*.^17^, 125 from Kandoth *et al*.^13^, 212 from Lawrence *et al*.^15^, 194 from Pritchard *et al*.^16^, 114 from Rahman *et al*.^1^ and 124 from Vogelstein *et al*.^14^ Thirty-nine additional genes were included based on the analysis of driver mutations in publicly available TCGA data, the published guidelines for return of results of the American College of Genetics and Genomics^45^ and 18 novel cancer driver genes identified in recently published large-scale studies.

### Germline sites overlapping with recurrent somatic mutations

Recurrent somatic mutations were extracted from the high confidence filtered set of somatic mutations^13^ and germline variants overlapping them were further filtered to remove those having a reported global MAF>0.5% in the NHLBI Exomes (ESP6500SI-V2). Remaining variants were filtered to remove artifacts due to ambiguous alignments, simple repeats, reference sequence errors, putative somatic mutations in adjacent normal tissue, somatic mutations associated with clonal expansion in blood^46^ and variants with a VAF<10% in tumour or normal. No germline mutations were found to overlap somatic mutations in the same individual.

In addition to sites described in the main text, several rare germline variants overlapping somatic mutations in genes associated with toxin metabolism were also identified. This included three cases carrying *CYP2D6* (H352R), as well as one carrier of *ABCC2* (E943K; rs3740065). Variants in both genes have been reported to be associated with poor outcome in post-menopausal women treated with tamoxifen^47^,^48^ but their association with cancer predisposition remains undetermined. In addition, a germline variant at somatic R423Q site was found in the *CARD11* oncogene^49^ and another germline variant S650L in *PDGFRB* was identified. Interestingly, a *FLT3* germline variant (R387Q) was identified to have an overlapping somatic mutation in endometrial cancer.

### Identifying significant genes using burden tests

We determined the MAF cutoff for rare variants as 0.05% based on balancing the inclusion of possible false-positives versus the loss of possible true-positives in subsequent burden test and LOH analysis. For example, if one presumes that *P* values ⩽0.01 have a reasonable possibility of being retained as significant in a multiple hypothesis test, the 0.05 threshold only excludes 2 such points out of a total of 47 for *BRCA1* and 1 such point out of a total of 52 for *BRCA2*. Conversely, it excludes 24 points in the MAF range up to 1% that are very unlikely to show significance. Points having MAF>1% are likewise not likely to be of interest (Supplementary Fig. 2).

Burden test analysis was performed by comparing the frequency of rare germline truncation mutations in cancer-associated genes from the Pan-Cancer 12 germline data set (from 12 cancer types; cohort size=4,034) with WHI 1,039 control samples and those downloaded from the NHLBI ESP (6,503 including 2,203 African-Americans and 4,300 European-Americans unrelated individuals). Variant calling on the TCGA and WHI data set was done as previously described in the Methods section. Variants for the ESP 6,503, along with their minor allele frequency were downloaded from http://evs.gs.washington.edu/EVS/). The truncation variants (nonsense, splice_site, and frameshift indels) from both groups were limited to a list of genes previously associated with cancer (see cancer-associated genes section). Further filtering includes retaining variants with <1% minor allele frequency from 1000 Genomes Project and <1% cohort frequency in each cancer type. A pooled minor allele frequency (the average minor allele frequency of each variant between the test and control group) was calculated for each variant and only those whose pooled minor allele frequency was <0.05% were kept for burden analysis. We excluded events having insufficient numbers of observations, defined here as fewer than three in the combined cases and controls for the ESP cohort and fewer than two in the WHI cohort. We subjected the data to the TFT, evaluating the one-tailed *P* value in each case (observations significantly greater than controls). For reference, we also evaluated the data using the cohort allelic sum test, although these results were not carried forward for analysis, because they correlate with TFT. The TFT probabilities were then ranked by the standard FDR. This procedure was performed for each cancer type versus the control group. In addition, an overall burden test was performed for Pan-Cancer 12 germline data set versus the control group. A FDR cutoff of 10% for the Pan-Cancer 12 germline data set was used.

### Statistical methods of LOH analysis

Next-generation sequencing provides direct read counts of reference and variant alleles and each pair of counts comprises an observational sample of the actual variant allele fraction (VAFs) at its site. We devised several statistical procedures using these counts to test for allelic enrichment at sites within a subset of genes hypothesized to be relevant across cancer types and, moreover, to test the genes themselves for significant content of such sites. This is one component of a larger method to assess loss-of-function alleles in these genes.

The evaluation at each tumour variant site (truncation or missense) is based on two complementary aspects related to its VAF: (1) whether it is significantly higher than the VAF at its corresponding site in the matched normal sample and (2) whether it is significantly higher than the characteristic VAF in the general population of genes having somatic mutations. The first aspect was implemented using Fisher's exact test^50^ on a 2 × 2 table of allele type (reference and variant) versus sample type (tumour and normal). For the second test, we permuted all combinations of reference counts and variant counts of the somatic events for all other genes, thus obtaining a null distribution that can be used for computing tailed *P* values.

Each of these two calculations uses some component of unique information not available to the other: they are essentially independent tests of the same hypothesis. We used a standard transformation method from the mathematical statistics literature to combine these values into a single, overall result^51^. The list of *P* values for the entire complement of tested sites was then corrected for multiple hypothesis testing bias and ranked using the standard Benjamini–Hochberg FDR calculation^52^.

For the second type of test at the gene level, we took the following approach for truncation events. All mutated sites for the candidate gene were catalogued, as were all sites outside of that gene, the latter representing the mutation ‘background'. The statistical difference between the two sets was then calculated using a standard difference-of-means *t*-test on the tumour VAFs of the two groups, where the number of degrees of freedom is 2 less than the total number of sites in the test. This procedure was repeated for each gene-of-interest, after which multiple testing correction was again applied in the context of FDR. With respect to missense events, we found this procedure was not sufficiently sensitive, so we used an alternative test based on comparing the fraction of missense sites within each gene that showed significant LOH on the individual level to the corresponding fraction in a background set consisting of the genes from burden testing that did not show significant LOH for truncations. To minimize noise, we adopted somewhat strict criteria for this particular test: to be tallied as LOH, a site must have had a maximum of 1% FDR in the site test and we only tested genes that satisfied the following inclusion criteria: a minimum difference of fractional values of two percentage points and at least three events showing LOH at the 1% FDR level. We then applied Fisher's exact test on 2 × 2 tables of missense type (significant LOH and no discernable imbalance) versus cohort (test gene and background population), after which FDR was once again applied to the result.

An important aspect of the above methods is pre-conditioning of inputs. Previous studies^53^ have discarded sites based on their inability to attain a significant *P* value under the test being used, pointing out incidentally that excluding sites directly improves FDR. The latter observation is undoubtedly true, but this view misses the importance of the confidence level associated with a VAF estimate, as determined by the size of the sample used for its computation. Because of depth variations both between samples and within samples, the reliability (confidence) of VAF estimates as calculated from read counts varies from site to site. A specified CI for each VAF furnish is a rigorous metric on which reliability can be assessed and low-reliability points subsequently excluded from analysis. Because VAFs can approach the extremes of 0 and 1 and are also sometimes based on only 10 or 15 reads, the standard interval from sampling theory is not particularly useful. Instead, we used Wilson's interval^54^, which does not suffer appreciably in these circumstances. We chose an interval of 90% confidence (*Z*-score of ∼1.65), removing events whose larger distance (above or below the calculated VAF) exceeded 12%. The remaining ‘high-quality' data were then used in the tests described above. Results having FDR ≤20% were prioritized as significant.

### LOH analysis of germline truncations and missense variants

We applied our LOH analysis method as a refinement step to the burden analysis. Specifically, we tested all sites (and by extension the genes containing those sites) that burden testing identified as being significant, either in a Pan-Cancer context or as associated with a specific cancer type. Here we used a FDR of 15% to capture the widest set of genes that could be significant. In a sense, we used our LOH method as a ‘confidence filter' situated on top of burden analysis to eliminate false-positives. With oncogenes removed, the list of candidates at this stage consisted of 32 genes, including *ATM, BRCA1, BRCA2, BRIP1, MSH6* and *RAD51C* (the ‘burden test genes'). We also separated missense from nonsense alterations, the latter typically resulting in truncated, non-functional protein products and analysed these sets separately.

The statistical procedure outlined above is straightforward, but can be applied in various ways. For assessing the burden test genes, we selected each one individually and constructed the corresponding null distribution from all remaining non-burden test genes. That is, we excluded from the null all those genes for which there was already some evidence of possible significance. The same principle applied to testing individual sites: no variants from burden test genes were included in the null distributions.

### Calculation of hotspots of significant LOH

The calculation method for LOH discussed above identifies instances where the observed VAF in the tumour is higher than what is attributable to chance. Building on this, we now describe a subsequent calculation that identifies groups of such instances that are clustered spatially. These groupings are so-called ‘hotspots of significant LOH' and signal likely biological relevance. The null hypothesis is that instances of LOH, whether statistically significant or not, are distributed randomly. Since we are primarily interested in discovery, test regions are implemented as unbiased ‘sliding windows' rather than as specific domains, linkers and so on. A relevant LOH observation must satisfy two conditions:

- condition *A*: the LOH is statistically significant, as described above
- condition *B*: the LOH resides within the current test window

Status and spatial placement are independent of one another, meaning that the Bernoulli probability of a single LOH observation can be calculated as


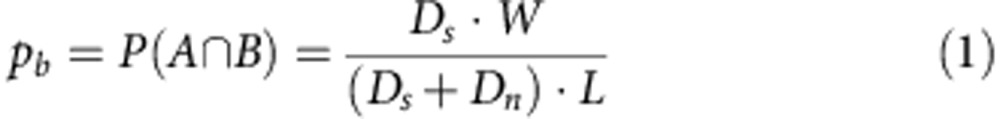


where *W* and *L* are the sizes of the test window and protein, respectively (in units of amino acids) and *D*_*s*_ and *D*_*n*_ are the total numbers of significant and non-significant LOHs observed for the protein. This expression indicates observations are of greater weight to the degree that the significant LOHs are more rare (as compared to non-significant LOHs) in the test set and that they cluster within tighter regions. LOHs are independently and identically distributed under the null hypothesis, meaning the mass probability of *k* observations of significant LOH within the window is then


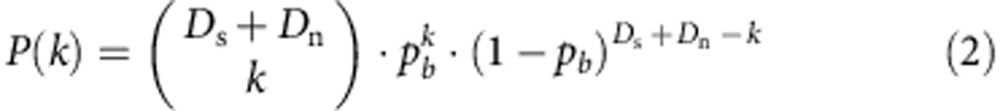


and the significance (test) probability of *k* observations within a test window is


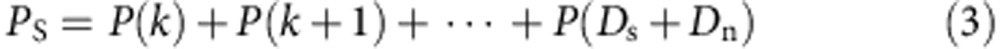


Since *p*_*b*_ is constant over a given protein for a given window size, appreciable caching can be used to economize the calculation. We use a slide-step of 1 amino acid and scan window sizes from 30 to 200, taking regions of significance to be characterized by their smallest *P*_S_. The software automatically merges overlapping significant regions. Standard FDR analysis, as described above, is then performed on the resulting list of hotspots.

### Functional validation of BRCA1 variants

Variants were incorporated into a full-length *BRCA1* expression plasmid, pcDNA-5′HA-BRCA1, using Q5 site-directed mutagenesis kit (New England BioLabs). Primer sequences are available in Supplementary Data 21. All of the desired variants were confirmed by sequencing.

HeLa-DR cells, a stable derivative of HeLa cells containing the genomic integration of the recombination substrate vector, pDR–GFP were used for the homology-directed recombination assay. Co-transfection of HeLa-DR cells with the *BRCA1* expression plasmid containing the test variant and siRNA targeting the 3′-untranslated region of the *BRCA1* gene to deplete endogenous BRCA1 expression was performed. Two days later, cells were transfected again with the siRNA, BRCA1 expression plasmid and the I-SceI expression plasmid. After 3 days, cells were harvested by trypsinization and the fraction of GFP-positive cells was determined using a FACScalibur flow cytometer (BD Biosciences model E1202). The plasmids and cell line used in this study have been described previously^31^.

All *BRCA1* variants were tested in triplicate and the percentage of cells with GFP expression was normalized to the rescue by wild-type BRCA1 expression plasmid.

## Additional information

**How to cite this article:** Lu, C. *et al*. Patterns and Functional Implications of Rare Germline Variants across 12 Cancer Types. *Nat. Commun.* 6:10086 doi: 10.1038/ncomms10086 (2015).

## Supplementary Material

Supplementary Information Supplementary Figures 1-6, Supplementary Note 1 and Supplementary References

Supplementary Data 1 Coverage and variant calling stats for discovery and control cohorts.

Supplementary Data 2 Summary of germline truncation variants identified in 624 cancer associated genes. Rare germline truncation variants (<0.05% MAF in discovery case and control combined) identified in 624 cancer associated genes across 4,034 cancer cases.

Supplementary Data 3 Rare germline truncation variants (<0.05% MAF in case and control combined) identified in 32 genes of interest across 1,627 validation cancer cases.

Supplementary Data 4 69 germline truncations validated using whole genome sequencing data.

Supplementary Data 5 Cancer associated gene lists used in this study, including 624 cancer associated genes, 114 cancer susceptibility genes reported in Rahman et al., 47 genes from Fanconi Anemia pathway.

Supplementary Data 6 Frequencies of rare truncation variants in 3 gene lists across 12 cancer types.

Supplementary Data 7 Burden analysis results for Pan-Cancer discovery cohort using rare truncation variants from 624 cancer associated genes in 3,125 Caucasian samples. The control cohort is 1039 WHI Caucasian cases.

Supplementary Data 8 Burden analysis results for Pan-Cancer discovery cohort using rare truncation variants from 624 cancer associated genes in 4,034 cases. The control cohort is ESP 6503 sample set.

Supplementary Data 9 Gene-based LOH analysis using rare truncation variants in significant genes from burden analysis.

Supplementary Data 10 Site-based LOH analysis for rare truncation variants in 624 cancer associated genes.

Supplementary Data 11 Gene-based LOH analysis for rare missense variants in 624 cancer associated genes.

Supplementary Data 12 Site-based LOH analysis for rare missense variants in 624 cancer associated genes.

Supplementary Data 13 LOH analysis of rare missense variants for discovering hotspot clusters.

Supplementary Data 14 Somatic mutations discovered in 3,368 out of 4,034 cancer cases.

Supplementary Data 15 Somatic and Germline Mutation Relationship (mutual exclusive/co-occuring) across 12 cancer types. The genes used are 34 burden test significant genes and recurrent mutated genes (>= 5 somatic mutations across all cancer types).

Supplementary Data 16 Somatic and Germline Mutation Relationship (mutual exclusive/co-occuring) for individual cancer types. The genes used are 34 burden test significant genes and recurrent mutated genes (>=2 somatic mutations in particular cancer type).

Supplementary Data 17 Distribution of BRCA1, BRCA2, and ATM germline truncation variants and somatic mutations across 12 cancer types.

Supplementary Data 18 Genes with rare germline truncation variants associated with somatic mutation frequencies.

Supplementary Data 19 Genes with rare germline truncation variants associated with younger age of initial diagnosis with cancer type as a covariate.

Supplementary Data 20 Genes with rare germline truncation variants associated with younger age of initial diagnosis for each cancer type.

Supplementary Data 21 Primers used for creating 72 BRCA1 expression constructs with 68 rare missense variants introduced and 4 control truncation constructs.

Supplementary Data 22 Homologous directed recombination assay results for 68 missense constructs, and 4 truncations as positive controls.

Supplementary Data 23 Summary of BRCA1 validation status for A.I./non A.I. events and the enrichment factor.

Supplementary Data 24 Rare germline missense variants overlapping with recurrent somatic mutations.

## Figures and Tables

**Table 1:**
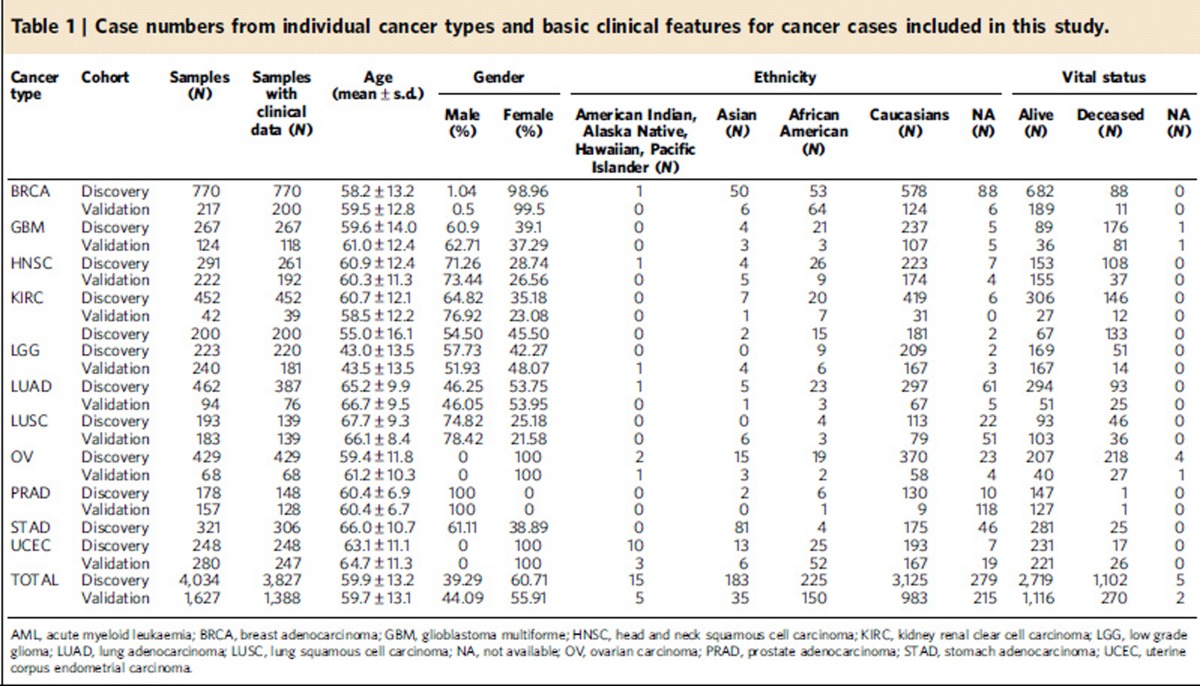
Case numbers from individual cancer types and basic clinical features for cancer cases included in this study.
