# Supplementary material for: Patterns and functional implications of rare germline variants across 12 cancer types
Source: Nat Commun. 2015 Dec 22;6:10086. doi: 10.1038/ncomms10086 (PMC4703835; doi:10.1038/ncomms10086)
Supplement: Supplementary Information — Supplementary Figures 1-6, Supplementary Note 1 and Supplementary References [file ncomms10086-s1.pdf]

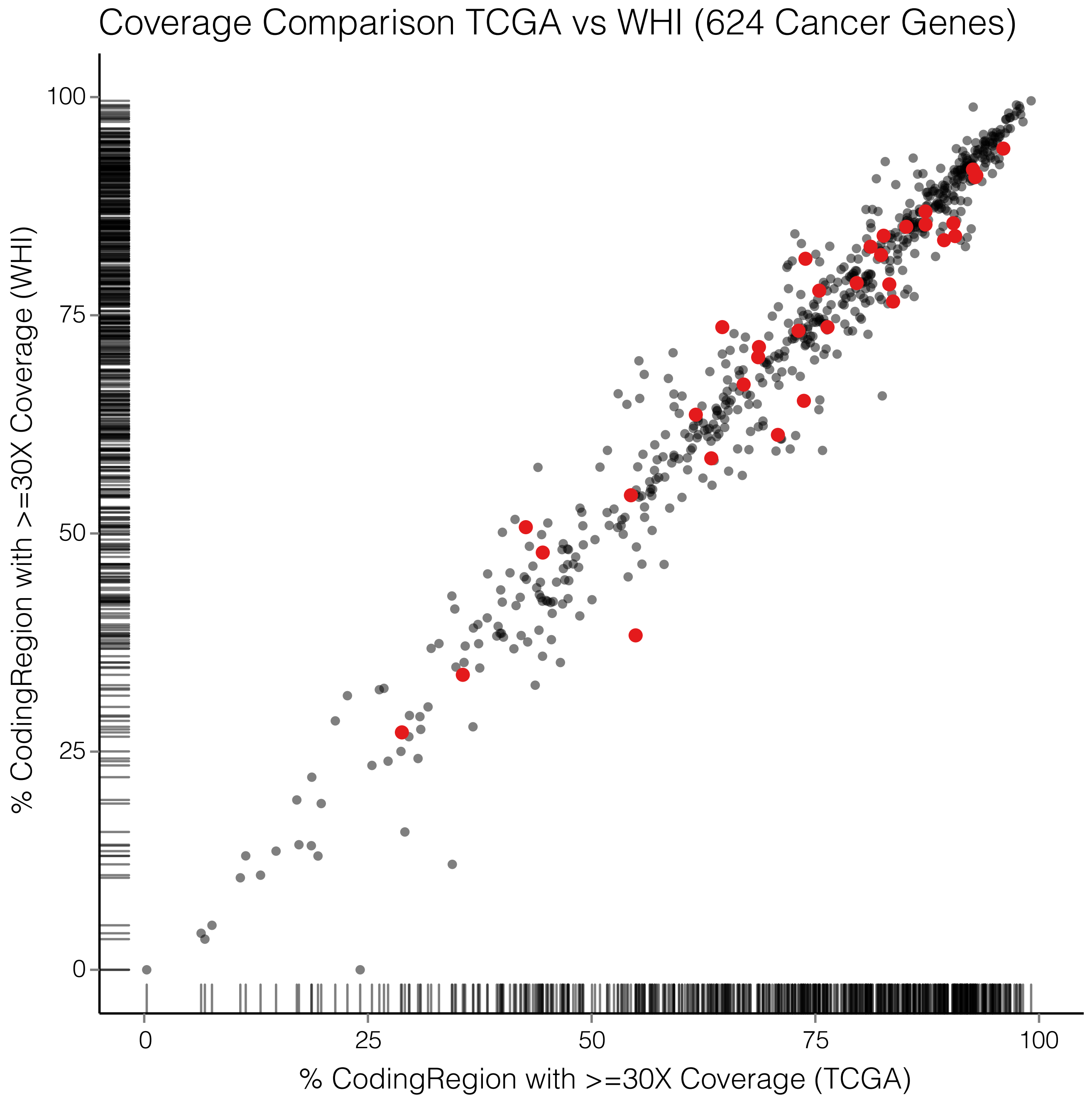

Supplementary Figure 1. Comparison of Coverage between the Caucasian TCGA cohort and WHISP cohort. We compared the coverage of the 624 cancer genes between 3,125 TCGA Caucasian and 1,039 WHI cases. The scatter plot shows the mean percent of coding regions with ≥30X coverage for each of these 624 genes in the TCGA cohort (X axis) and WHI (Y axis), with the Pearson's correlation coefficient of 0.98. A subset of the 624 dots (denoted in red) represents genes with significant enrichment of rare truncation variants determined in the TCGA cohort by the burden analysis.

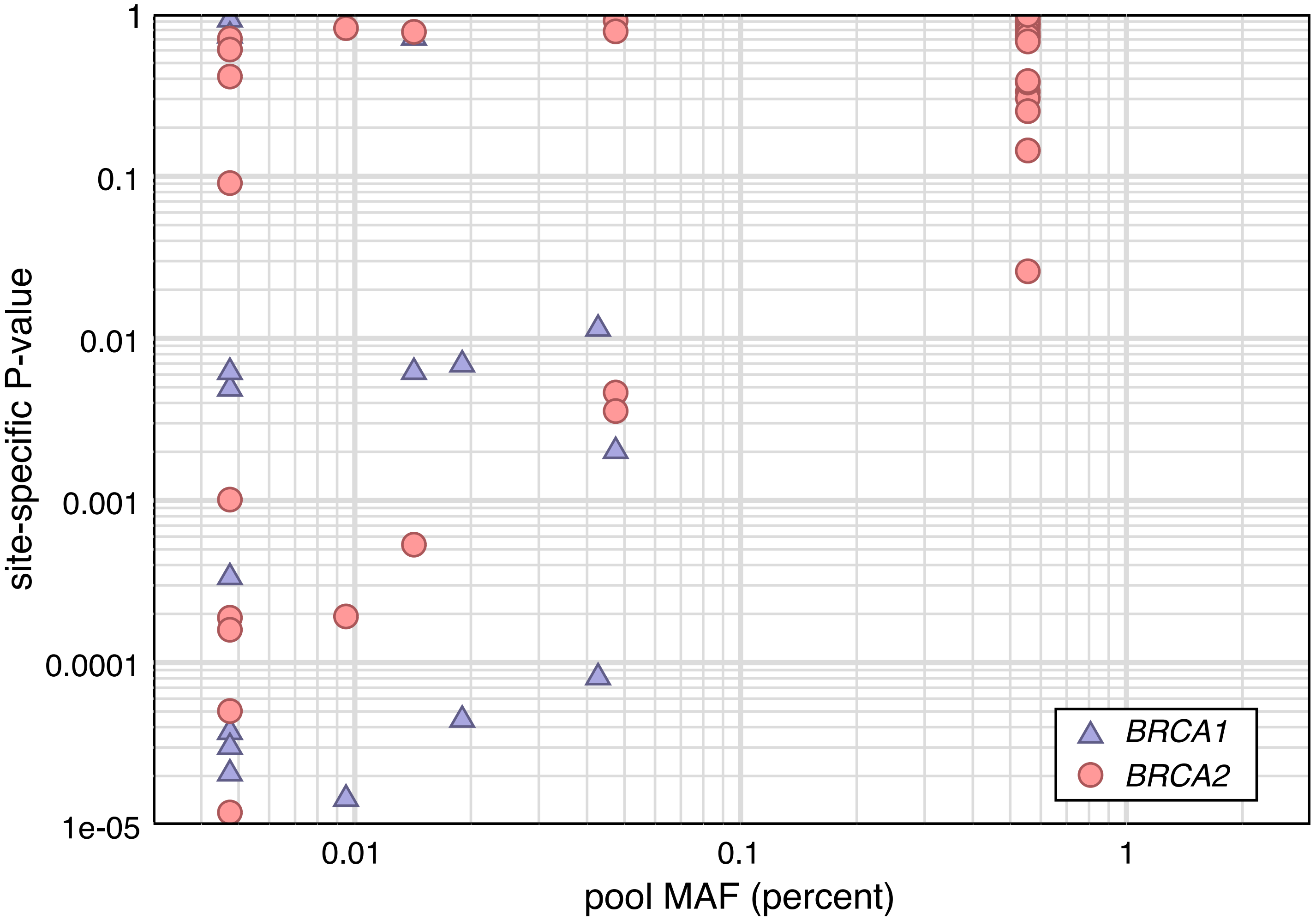

Supplementary Figure 2. Minor allele frequency analysis of BRCA1 and BRCA2 rare germline truncations. The scatter plot shows the relationship between MAFs of BRCA1 and BRCA2 germline truncation variants and site-specific P-values from LOH analysis. For both BRCA1 and BRCA2, the variants with low MAFs tend to display significant LOH. Selecting 0.05% cutoff for rare variants was based on balancing the inclusion of possible false-positives versus the loss of possible true-positives in subsequent burden test and LOH analysis.

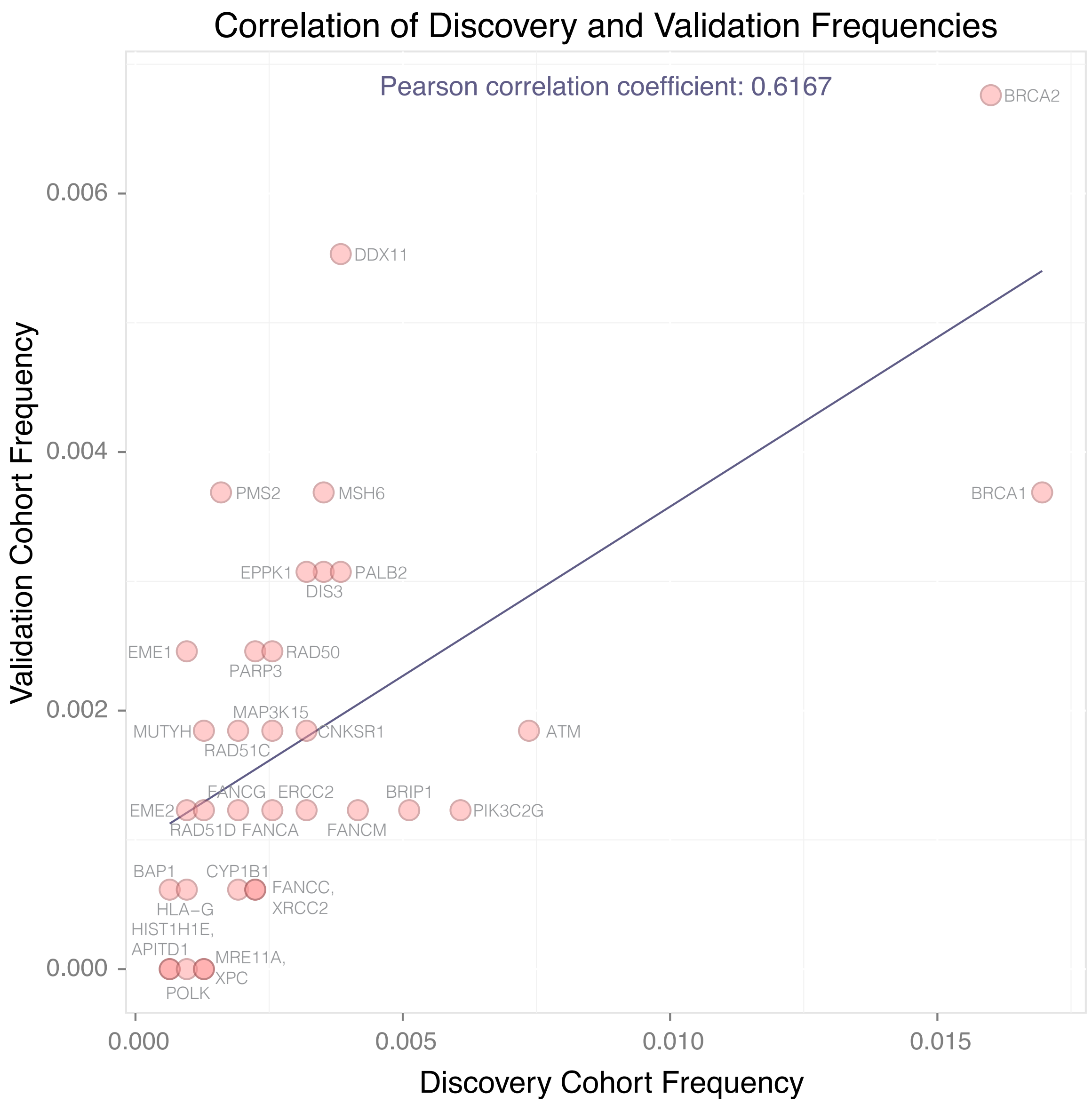

Supplementary Figure 3. Correlation of truncation frequencies in 32 genes of interest between discovery set and validation set. Using the 3,125 and 1,627 cases in the discovery and validation cohorts, respectively, we used gene-specific tallies to calculate frequencies of truncations in the 32 genes found to be significant by burden testing. Figure shows the discovery and validation frequencies for each gene plotted on the abscissa and ordinate, respectively. These data were regressed using the ordinary least-squares (OLS) calculation. A Pearson's correlation coefficient of 0.6167 was found and the resulting regression line is also plotted (red).

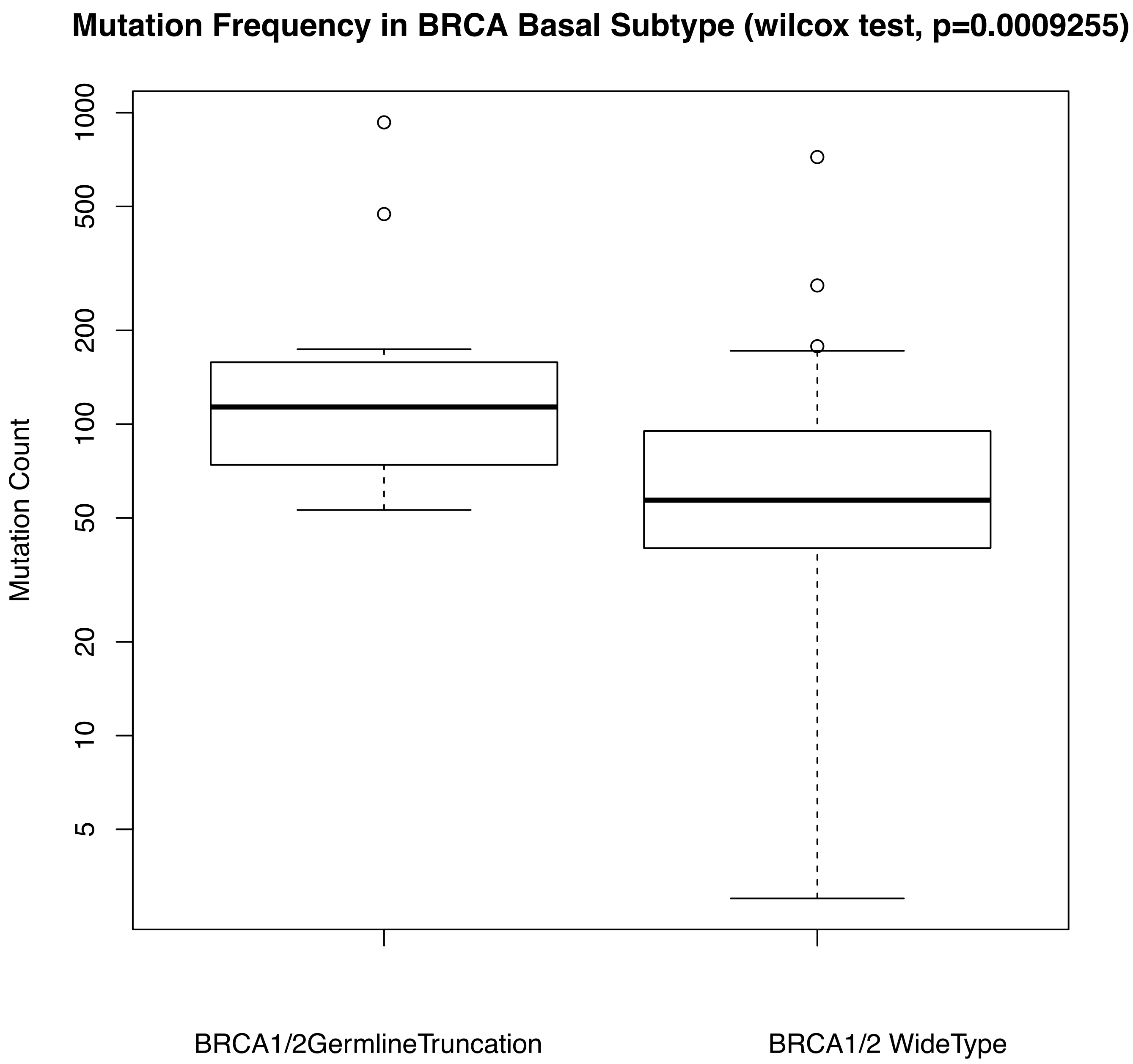

Supplementary Figure 4. Mutation rate comparison within BRCA basal subtype. Boxplot shows the mutation rate distribution in basal cases with BRCA1/2 rare germline truncation (n=12) and basal cases without BRCA1/2 rare germline truncation variants (n=101, 2 cases missing somatic mutation information). Graph shows median central line, 50% confidence interval box and 95% confidence interval whiskers. P-value is calculated by Wilcoxon rank-sum test.

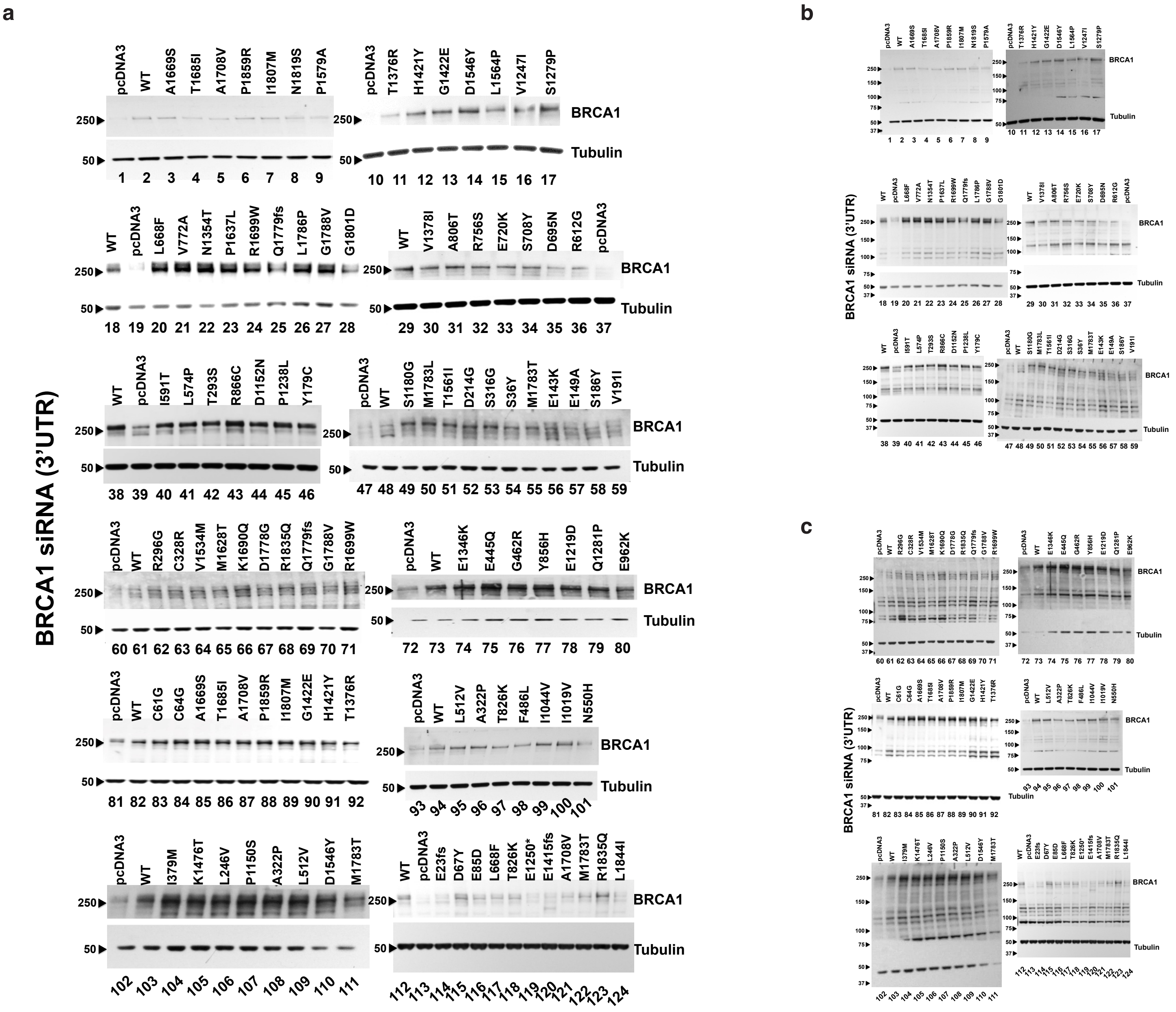

Supplementary Figure 5. Western blot analysis of expression of BRCA1 mutant constructs. a) HDR assay results were tested for protein expression of the BRCA1 variant from the transfected plasmid. Cells that remained following flow cytometry analysis were extracted and soluble proteins analyzed by immunoblots. Since multiple experiments were done, each panel should be compared separately from the others. In all samples, the endogenous BRCA1 protein had been depleted by transfection of the siRNA targeting the 3'-UTR of the BRCA1 mRNA. All blots were probed for BRCA1, and strips show the proteins that migrated at about the 250 kDa marker, and strips were probed for a-tubulin, which migrated near the 50 kDa marker, as a loading control. Samples from the various BRCA1 plasmid transfections were as follows: vector only (lanes 1, 10, 19, 37, 39, 47, 60, 72, 81, 93, 102, 113); WT (lanes 2, 18, 29, 38, 48, 61, 73, 82, 94, 103, 112); E23fs (lane 114); S36Y (lane 54); C61G (lane 83); C64G (lane 84); D67Y (lane 115); E85D (lane 116); E143K (lane 56); E149A (lane 57); Y179C (lane 46); S186Y (lane 58); V191I (lane 59); D214G (lane 52); L246V (lane 106); T293S (lane 42); R296G (lane 62); S316G (lane 53); A322P (lanes 96, 108); C328R (lane 63); I379M (lane 104); E445Q (lane 75); G462R (lane 76); F486L (lane 98); L512V (lanes 95, 109); N550H (lane 101); L574P (lane 41); I591T (lane 40); R612G (lane 36); L668F (lanes 20, 117); D695N (lane 35); S708Y (lane 34); E720K (lane 33); R756S (lane 32); V772A (lane 21); A806T (lane 31), T826K (lanes 97, 118); Y856H (lane 77); R866C (lane 43); E962K (lane 80), I1019V (lane 100); I1044V (lane 99); P1150S (lane 107); D1152N (lane 44); S1180G (lane 49); E1219D (lane 78); P1238L (lane 45); V1247I (lane 16); E1250* (lane 119); S1279P (lane 17); Q1281P (lane 79); E1346K (lane 74); N1354T (lane 22); T1376R (lanes 11, 92); V1378I (lane 30); E1415fs (lane 120); H1421Y (lanes 12, 91); G1422E (lanes 13, 90); K1476T (lane 105); V1534M (lane 64); D1546Y (lanes 14, 110); T1561I (lane 51); L1564P (lane 15); P1579A (lane 9); M1628T (lane 65); P1637L (lane 23); A1669S (lanes 3, 85); T1685I (lanes 4, 86); K1690Q (lane 66); R1699W (lanes 24, 71); A1708V (lanes 5, 87, 121); D1778G (lane 67); Q1779fs (lanes 25, 69); M1783L (lane 50); M1783T (lanes 55, 111, 122); L1786P (lane 26); G1788V (lanes 27, 70); G1801D (lane 28); I1807M (lanes 7, 89); N1819S (lane 8); R1835Q (lanes 68, 123); L1844I (lane 124); and P1859R (lanes 6, 88). b,c) Uncropped images.

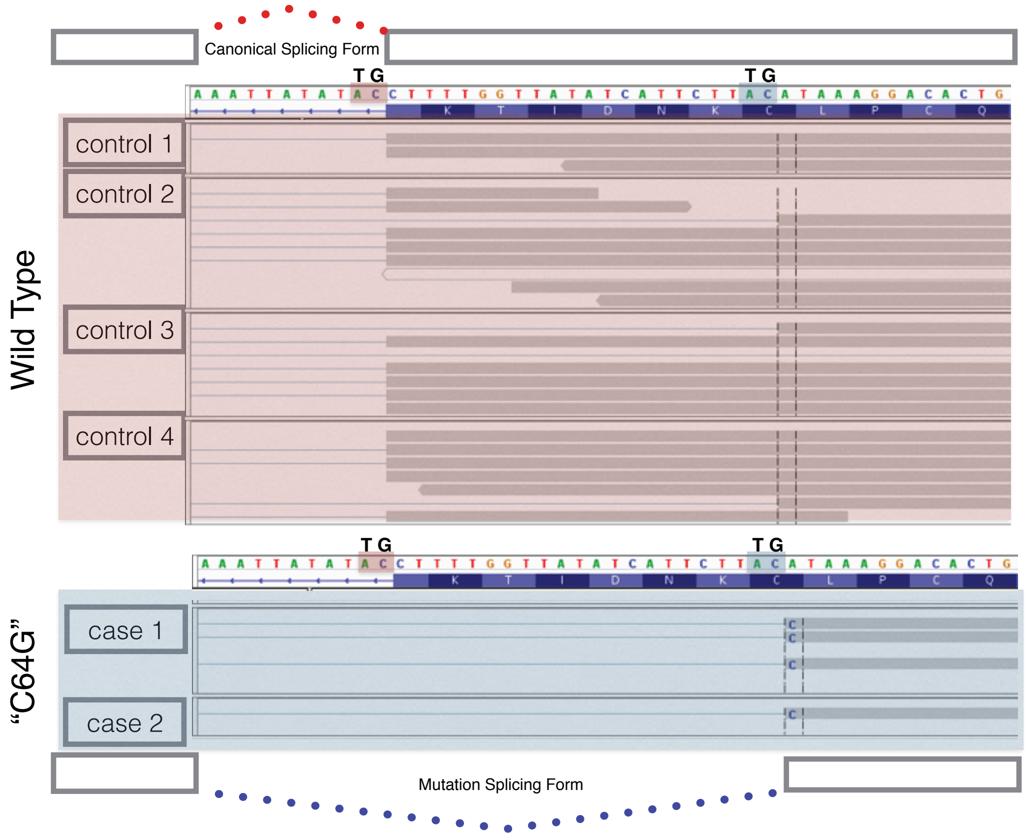

Supplementary Figure 6. Impact of C64G mutation in BRCA1 on splicing. Integrated genomic viewer screen capture of C64G mutation, leading to the activation of an infrequently used splice site, is shown. Two ovarian cases with C64G mutation and four ovarian cases without this mutation were shown.

Supplementary Note 1

We also examined germline sites overlapping recurrent somatic mutations found in the 12 TCGA cancer types[1] (**Supplementary Data 24**). After stringent filtering, we identified 34 missense germline hotspot variants that overlapped recurrent somatic mutations in cancer associated genes, based on the somatic mutations reported in Kandoth *et al*[1]*.* Most of these 34 hotspot missense germline variants affected conserved nucleotide/amino acid residues. This list includes six variants in the DNA-binding domain of *TP53*, all occurring at five previously identified hotspots (R110H, R158C, R267Q, R175C, and G245V). G245V has been reported by the IARC as nonfunctional[2], while the four remaining variants were reported to have partial functionality (http://p53.iarc.fr/). One *ATM* (R2691C) variant, involved in CLL[3], is also known to interact with TP53 and can result in the transformation of the ATP binding pocket. Another prominent cluster of rare germline missense mutations appeared in DNA-repair (Fanconi Anemia) pathway. These included two recurrent variants (A625T) in *PARP1*, somatically mutated in bladder and endometrial cancer[1], a variant (E201K) in *DDX11*, proximal to a validated functional missense variant (R263Q) responsible for Warsaw Breakage[4], and one variant (R1084C) in *FANCA*. In addition, a germline variant (K140N) in the *BRCA1*-binding-partner *BARD1* was identified and its three adjacent residues were found to be recurrently mutated in multiple cancer types (COSMIC). A missense variant (E2020K) in *BRCA2*, recurrently mutated in other samples, is currently classified as a variant of unknown significance (BIC/Brody)[5].

Using existing clinical significance data from the NCBI ClinVar database (http://www.ncbi.nlm.nih.gov/clinvar/), a total of 101 rare germline missense variants were listed as Pathogenic. Of these, 40 were from tumor suppressor genes (TSG) or encoded proteins involved in DNA repair, including *POLH* (DNA repair; 9 variants), *BUB1B* (TSG; 9), *VHL* (TSG; 5), and *APC* (TSG; 5), among others. *BRCA2* was also on the list but had only 2 variants. The low occurrence of *BRCA2* variants and lack of *BRCA1* variants merit further investigation and suggest that this approach may be improved by querying additional clinical databases specific to breast cancer and other types of cancer. Other identified variants were involved in other diseases (e.g. *LRRK2* for Parkinson's disease) and also included an oncogene (*RET* proto-oncogene, 8) and DNA transcription factors (e.g. *AR*, 4). Additionally, only *TYR* and *BRCA2* are common to this list and the list of significant non-oncogenes obtained from truncation variant analysis, suggesting that the combination of both methods could be especially useful for identifying germline variants involved in cancer.

Supplementary References

1. Kandoth, C. *et al.* Mutational landscape and significance across 12 major cancer types. *Nature* **502**, 333-9 (2013).
2. Kato, S. *et al.* Understanding the function-structure and function-mutation relationships of p53 tumor suppressor protein by high-resolution missense mutation analysis. *Proceedings of the National Academy of Sciences of the United States of America* **100**, 8424-9 (2003).
3. Guarini, A. *et al.* ATM gene alterations in chronic lymphocytic leukemia patients induce a distinct gene expression profile and predict disease progression. *Haematologica* **97**, 47-55 (2012).
4. Capo-Chichi, J.M. *et al.* Identification and biochemical characterization of a novel mutation in DDX11 causing Warsaw breakage syndrome. *Human mutation* **34**, 103-7 (2013).
5. Szabo, C., Masiello, A., Ryan, J.F. & Brody, L.C. The breast cancer information core: database design, structure, and scope. *Human mutation* **16**, 123-31 (2000).
